# Treatment of Frey Syndrome with Botulinum Toxin-A: A Practical Approach from Minor’s Test to Injection

**DOI:** 10.1007/s12663-023-02029-9

**Published:** 2023-11-09

**Authors:** Lukas S. Fiedler, Fabian Burk

**Affiliations:** 1 Department of Otorhinolaryngology and Head and Neck Surgery, University Medical Center Hamburg-Eppendorf, Hamburg, Germany; 2 Department of Phoniatrics and Pedaudiology, University Medical Centre Münster, Münster, Germany

**Keywords:** Frey syndrome, Treatment, Botox, Botulinum toxin, Minor starch-iodine test, Parotidectomy, Gustatory sweating

## Abstract

**Introduction:**

Frey's syndrome, described by Lucy Frey in 1923, is a unique condition characterized by sweating, flushing, and reddening as a direct response to mastication. This phenomenon results from the aberrant regeneration of postganglionic parasympathetic neurons originating from the auriculotemporal nerve and the subsequent acetylcholine secretion induced by masticatory stimuli. Although rare, this syndrome can have multiple underlying causes and is frequently observed, occurring in up to 65% of cases following lateral parotid resections. Additionally, it can less commonly manifest after neck dissection, facelift procedures, or be associated with diabetes mellitus.

**Method:**

This article outlines a comprehensive diagnostic algorithm for Frey's syndrome, which includes the utilization of the Minor-Starch-Iodine Test. This test is a key component in diagnosing the syndrome and is discussed in detail, providing insights into its procedure and interpretation. Additionally, the gold standard of treatment for established Frey's syndrome, botulinum toxin A, is thoroughly described, including its mechanism of action, administration, and potential side effects.

**Discussion:**

Finally, the article underscores the need for further research to enhance our understanding of Frey's syndrome, leading to better diagnostic methods and more tailored treatment options for patients.

## Introduction

Frey syndrome (FS), also known as auriculotemporal syndrome or gustatory sweating can occur as a consequence of surgical intervention or trauma in the parotid region. It is due to aberrant reinnervation after trauma to postganglionic nerve fibers of the auriculotemporal nerve to postganglionic parasympathetic neurons to the surrounding denervated sweat glands and cutaneous blood vessels. As a result, vasodilatation and activation of sweat-glands are triggered by nerval stimuli—namely the secretion of acetylcholine (ACH)—which are salivatory in their original intent. Thus, reddening, warming and sweating ensue in the affected skin areas. The severity of symptoms can range from virtually asymptomatic cases to serious impairment of quality of life.

Albeit multiple surgical treatment methods for manifest FS have been described, intradermal (ona)botulinum toxin-A (BTX) injection of the affected areas determined by the Minor starch-iodine test (Msit) represents an effective, symptom-oriented, hardly invasive, repeatable and easy-to-learn treatment method [1]. Patients normally show improvement after 4–7 days and the BTX- therapy normally hinders symptom recurrence for 7–10 months [2]. Due to the authors experience FS symptoms decrease after repeated BTX injections. As comprehensive overview is not the scope of this technical note, we recommend the article by Young and Okuyemi [3] as a starting point for further background information, prevention and treatment techniques and literature.

## Minor Starch-Iodine Test

To identify the affected skin areas the MSIT is performed.

**Fig. 1:**
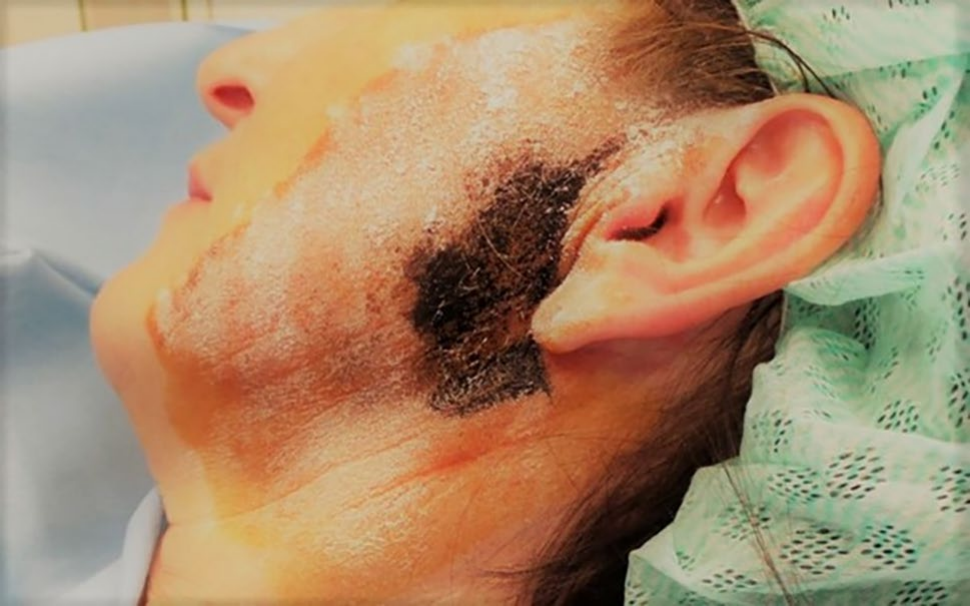
Marked left parotid area after Minor starch-iodine test

Firstly, iodine solution, e.g. 5% Lugol’s solution (Iodine-potassium-iodide) is applied to the skin area indicated by anamnesis. After letting it dry out completely, starch powder is applied to the same area. Then, an adequate salivatory stimulus, a slice of lemon for example, is provided orally. In manifest FS, sweating occurs in the affected area. Upon contact with moisture the iodine forms complexes with the amylose in the starch and the colour turns blue or black, marking the affected area. The borders are then outlined with a permanent marker and the area is cleaned (Fig. 1).

## Botulinum Toxin-A Injection

Always perform a thorough anamnesis, check for allergies and contraindications and get informed consent before administering this potent neurotoxin.

**Fig. 2:**
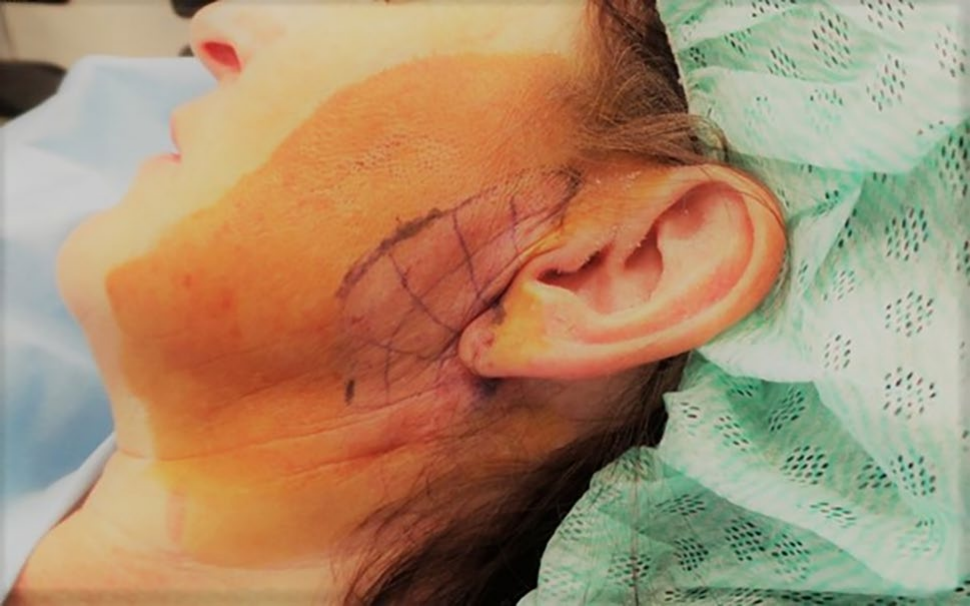
The affected area is divided into a grid of approximately 1 cm^2^ squares

For easier distribution of BTX the target area can be divided into a grid of 1 cm^2^ squares, reflecting the approximate intradermal diffusion distance of BTX. The area can be cooled to reduce discomfort due to the injections. After application of disinfectant, it is crucial to wait until the alcohol is completely evaporated so as not to risk deactivation of the toxin. In the meantime, the BTX, which comes in form of a powder is solved in 0.9% sodium-chloride. The ratio and the resulting volume must be chosen with respect to the number of injection-points (Fig. 2).

**Fig. 3:**
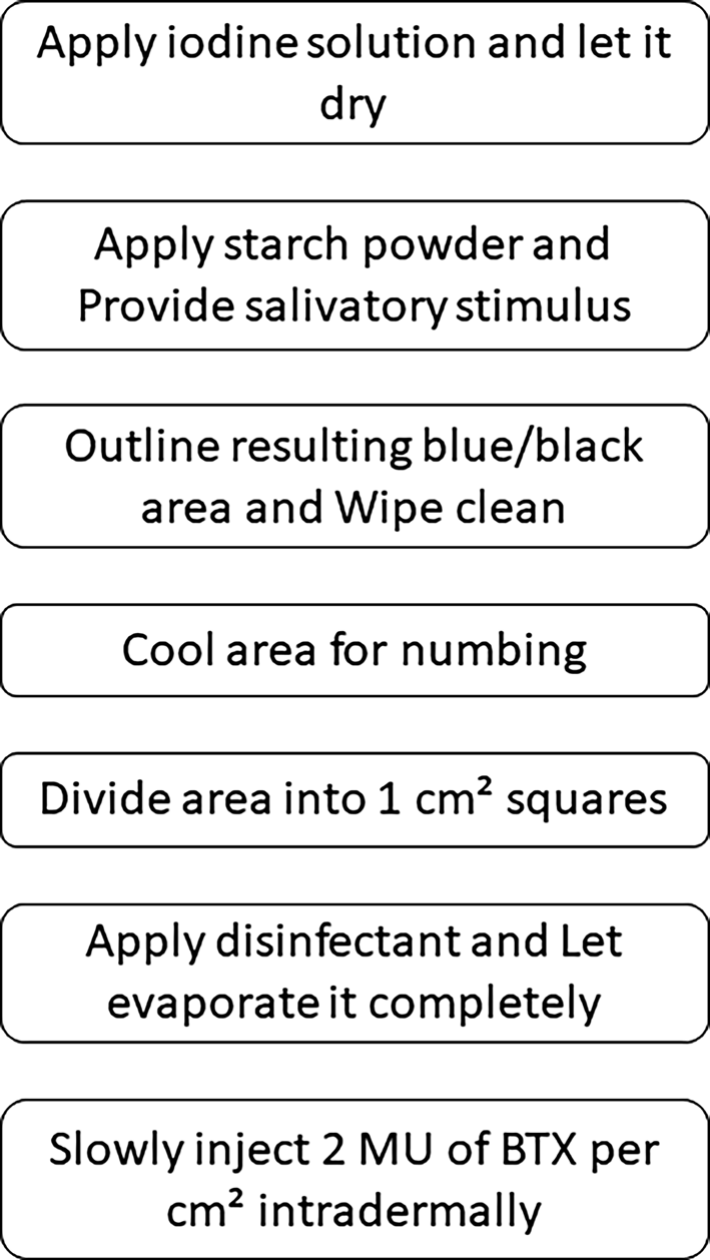
Procedure instructions (top to bottom) after the diagnosis of Frey’s syndrome is established, thorough patient history has been taken (including allergies and potential contraindications) and informed consent was given by the patient. BTX, botulinum toxin-A

A thin intradermal needle (26 gauge or higher) is used to inject two mouse units of BTX per 1 cm^2^ square. Here, the dosage is given for onabotulinum toxin-A (Botox®) and can vary depending on the used formulation. Keep a cotton swab at hand to stop minor bleeding at injection points. Upon entering the axon, the toxine cleaves the SNAP-25 protein preventing the release of ACH [4]. Strictly intradermal injection is important to avoid paralysis of underlying (e.g. mimic) muscles. Effects will become apparent not before 4–7 days. Because of SNAP-25 resynthesis in the axon, the effects last only a few months, typically about 7–9. The procedure can be repeated both in the case of residual and of recurrent symptoms (Fig. 3).
